# Association of hormone therapy with spheno-orbital meningiomas: bridging evidence and unknowns

**DOI:** 10.3389/fonc.2026.1764350

**Published:** 2026-03-16

**Authors:** Jarett E. Prince, Kivanc Yangi, Michell Goyal, Kashif Qureshi, Jack T. Olson, Egemen Gok, Mark C. Preul

**Affiliations:** The Loyal and Edith Davis Neurosurgical Research Laboratory, Barrow Neurological Institute, St. Joseph’s Hospital and Medical Center, Phoenix, AZ, United States

**Keywords:** cyproterone acetate, hormone therapy, meningioma, progesterone receptors, skull base tumors, spheno-orbital meningioma, surgical oncology, tumor regression

## Abstract

**Objective:**

To evaluate the association between hormone therapy and the development, progression, and treatment response of spheno-orbital meningiomas (SOMs) and to identify current evidence gaps in clinical management.

**Methods:**

A systematic literature review was conducted using an advanced search of PubMed, Embase, Scopus, and Cochrane databases for articles published in English. The selection process adhered to the PRISMA guidelines. Inclusion criteria targeted original research published from inception to May 2025, which discussed hormone therapy exposure and SOMs. Study quality was assessed with Joanna Briggs Institute critical appraisal tools appropriate to each study design.

**Results:**

Twenty articles were retrieved. After screening, 10 studies met the inclusion criteria and were analyzed. The studies comprised 2 case reports, 1 prospective cohort, and 7 retrospective cohorts, totaling 315 patients with SOMs. The gender distribution was predominantly female (166 women, 4 men) in the 7 studies that reported mean age data for patients with SOM. The patients had a mean (SD) history of 12.6 (3.67) years of hormone therapy exposure, with 90% of the therapies being progestins. Six studies reported a decrease in the SOM volume after hormone therapy cessation, and 4 studies documented a decrease in the soft tissue component with progression or stabilization of the intraosseous component. The therapeutic goal was surgical resection. Subtotal resection was associated with higher recurrence than total resection, especially when the residual tumor included soft tissue. Conservative management, involving hormone therapy cessation, was reported in 3 cases. Data were limited regarding progesterone receptor status and the use of radiation therapy.

**Conclusions:**

Current evidence suggests that hormone therapy, particularly long-term exposure to progestins, may contribute to the development and progression of SOMs. Although cessation of hormone therapy can result in partial tumor regression, especially in soft tissue components, surgical resection remains the primary treatment. Hybrid strategies that combine hormone therapy cessation with surgery may be beneficial in selected cases, although prospective data are lacking. Standardized clinical guidelines and further studies are needed to clarify the role of hormone therapy in the management of SOM.

## 1 Introduction

Spheno-orbital meningioma (SOM), first described by Harvey Cushing as an “en plaque” meningioma, is a rare and distinct subset of skull base tumors, accounting for up to 9% of all intracranial meningiomas (1–4). These tumors arise from the sphenoid wing and often extend posteriorly into the cavernous sinus and anteriorly into the orbital apex, creating complex anatomical challenges (1, 5, 6). SOMs characteristically involve both intraosseous and intradural soft tissue components, typically occurring as sphenoid wing hyperostosis with diffuse, carpet-like soft tissue infiltration (1, 6, 7). This infiltration leads to compression of orbital and neurovascular structures, with symptoms such as proptosis, progressive visual impairment, and, less frequently, oculomotor dysfunction (1, 3, 6, 7).

Surgical resection is the mainstay of treatment and is guided by a symptom-oriented paradigm to revert the neuro-ophthalmologic symptoms, with an emphasis on quality of life and functional outcomes (8). Multiple surgical approaches have been developed, including microsurgical transcranial, endoscopic endonasal, endoscopic transorbital, and combined approaches (9). Various forms of the microsurgical transcranial approach are the most commonly used (2, 9–12) and reportedly have the best rate of achieving gross total resection (9). However, accomplishing a gross total resection is often limited by the proximity of the tumor to critical neurovascular structures, and recurrence rates range from 35% to 50% (1). Notably, the extent of resection of the soft tissue component is more strongly associated with reduced recurrence rates than resection of the bony component alone (8, 13). Therefore, a nuanced pathophysiological understanding is important to guide effective surgical management and preoperative planning.

An increasing body of evidence suggests that hormonal factors contribute to meningioma pathogenesis (4, 14–16), supported by the high prevalence of progesterone receptors (PRs) in these tumors (15, 17, 18). Progesterone likely promotes meningioma growth by activating genes involved in cell growth. This effect may depend not just on the presence of PRs but also on steroid coactivators, which aid the receptors in propagating growth signals (19, 20). Notably, SOMs tend to be a *TRAF*_7_*/PIK*_3_*CA* subtype of meningioma, exhibiting high PR expression and enhanced sensitivity to exogenous progestins (21). Studies have shown both an elevated prevalence of SOMs in women receiving hormone therapy and high rates of PR positivity in tumor samples (4, 8, 14, 22, 23). Estrogen receptor positivity has also been linked to skull base meningiomas, although direct evidence in SOMs remains limited (4, 8, 14, 22, 23). Unfortunately, despite these findings and the growing interest in the hormonal sensitivity of SOMs, most available data are gathered from patients with all meningioma subtypes, with few cases focused on the spheno-orbital subtype (4, 24).

To our knowledge, no comprehensive review has explored the relationship between hormone therapy and SOM behavior. Moreover, no known clinical framework or guideline exists for managing hormone therapy in patients with SOMs. Therefore, we conducted a systematic literature review to identify clinical and treatment patterns, highlight knowledge gaps, and propose directions for future research and management.

## 2 Materials and methods

### 2.1 Search strategy

This study adhered to the Preferred Reporting Items for Systematic Reviews and Meta-Analyses (PRISMA) guidelines (25). To identify studies, we used the PubMed (MEDLINE), Embase (Elsevier), Scopus (Elsevier), and The Cochrane Library (John Wiley & Sons, Inc) databases. Systematic searches were conducted in May 2025 for publications from inception to 2025 using the following keywords: ((Hormone) OR (Hormonal therapy)) OR (Hormone therapy)) OR (Hormone replacement therapy)) OR (HRT)) OR (Progestin)) OR (Progestin therapy)) OR (Replacement Therapy, Hormone)) OR (Therapy, Hormone Replacement)) OR (Hormone Replacement Therapies)) OR (Replacement Therapies, Hormone)) OR (Therapies, Hormone Replacement)) AND ((Spheno-orbital meningioma) OR (Sphenoorbital meningioma)). Boolean operators AND and OR were used to combine these Medical Subject Headings terms, which created a comprehensive search strategy.

### 2.2 Eligibility criteria

This study aims to determine how exposure to exogenous hormone therapy, particularly progestins, influences tumor development, growth dynamics, and clinical outcomes in adult patients with SOMs, compared with those who have not received such hormonal exposure. Our inclusion criteria were original research articles that discussed an association between hormone therapy and SOMs. Articles were excluded if they lacked a discussion of hormone therapy and SOMs. Additionally, we excluded review articles, editorials, preprints, conference abstracts, unpublished studies, commentaries, retracted publications, errata, studies lacking accessible full texts, and articles unavailable in English.

### 2.3 Study selection

After receiving the results from our query, duplications were excluded, and English-language articles were screened for inclusion. The reference check method and a manual search were also conducted to identify additional articles that met our inclusion criteria. All articles were uploaded to the Rayyan platform (26) and screened by 3 independent reviewers (J.T.O., J.E.P., K.Y.). All reviewers agreed to include each article in this review.

### 2.4 Quality assessment

To assess the methodological quality and potential bias in the included studies, we applied the Joanna Briggs Institute (JBI) Critical Appraisal Checklists (https://doi.org/10.46658/JBIMES-20-08) (27). Case reports were evaluated using the JBI tool for case reports, and cohort studies were assessed with the JBI checklist for cohort studies (https://jbi.global/critical-appraisal-tools). These tools were selected for their structured approach to evaluating internal validity and relevance, tailored to each study design. Two independent reviewers (J.E.P., K.Q.) assessed the studies for each of the JBI questions. A third reviewer (M.G.) addressed any remaining unresolved disputes until agreement was reached. A summary of the assessments is presented in **Supplementary Table 1** (4, 12, 13, 22, 28–33).

## 3 Results

**Figure 1:**
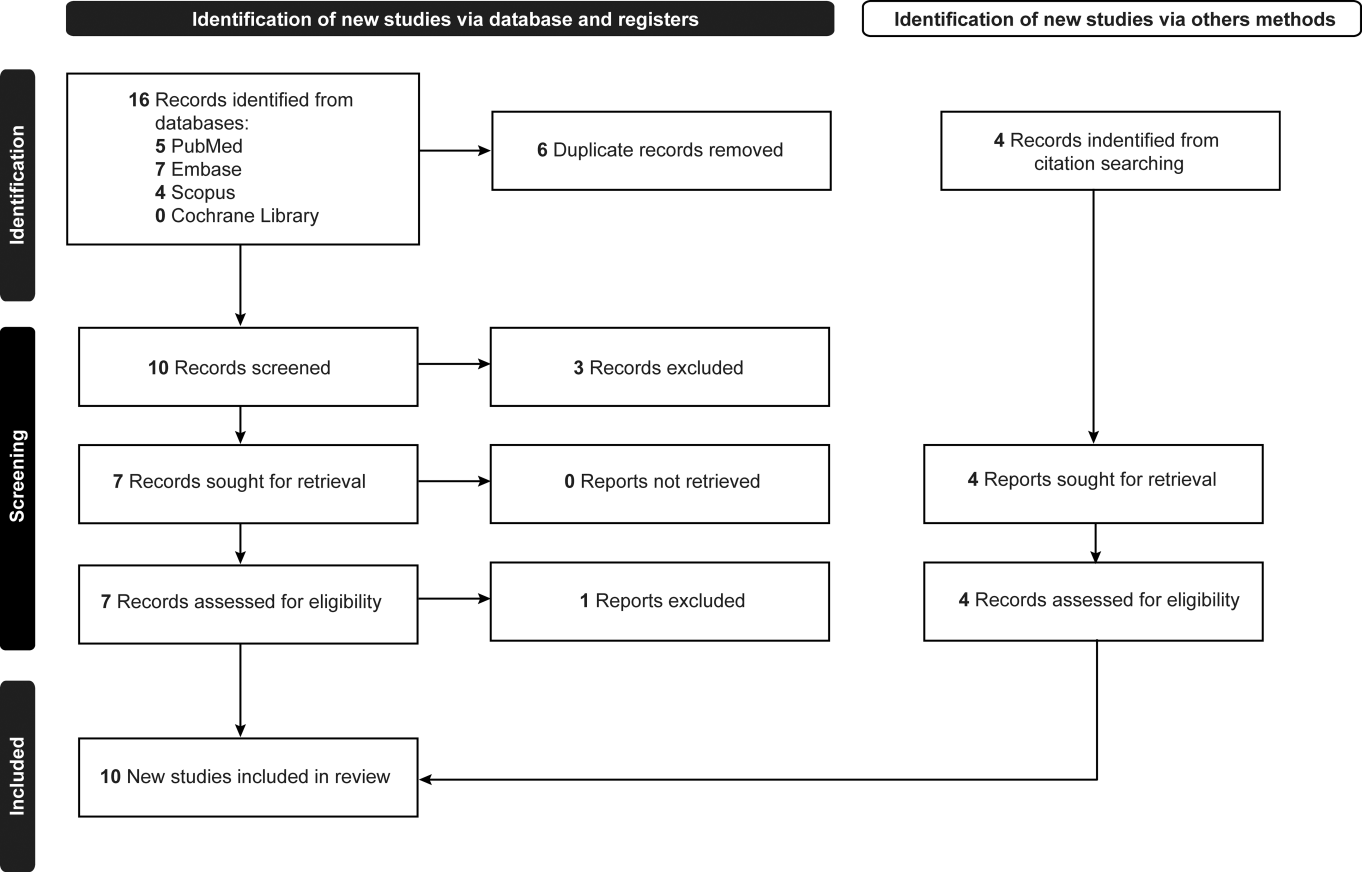
PRISMA (Preferred Reporting Items for Systematic Reviews and Meta-Analyses) flow diagram documenting the study selection process. *Used with permission from Barrow Neurological Institute, Phoenix, Arizona*.

Ten studies were included in this study (**Tables 1**, **2**) (4, 12, 13, 22, 28–33). They included 2 case reports, 1 prospective cohort, and 7 retrospective cohort studies. The selection process is documented in **Figure 1** (6, 12, 13, 22, 28, 29, 33, 34). Across the 10 studies, data were extracted from 315 patients. All included studies addressed patients with a diagnosis of SOMs. The gender distribution was predominantly female (166 women, 4 men) in the 7 studies that reported mean age data for patients with SOM (4, 12, 22, 28, 29, 32, 33). The remaining 3 studies reported the mean age for their overall meningioma cohorts (13, 30, 31) and were therefore excluded from the mean age calculation in our review. To avoid the bias introduced by averaging study means directly, we calculated a weighted mean age and standard deviation based on the number of patients with SOMs in each study. The weighted mean (SD) age of patients in our review was 50.0 (1.85) years (range of study means: 42.0–57.0). The median of the 7 study means was 49.5 years.

**Table 1:** Summary of studies focused on the relationship between hormone therapy and SOMs^a^.

Author	Study type	No. of patients by gender	Mean age at Dx, y	Pre-Dx hormone therapy use; reason	Mean duration of hormone therapy, y	Hormone therapy use on Dx?	Side of tumor	Hormone therapy cessation? when?
Apra et al., 2020 (22)	Retrospective cohort	4 M, 78 F	51.6^b^	Progestogenic therapies (41 patients), estroprogestogenic therapies (13), NS (28); varied reasons	10	Yes	NS	Yes; postop
AbiJaoude et al., 2021 (28)	Case report	1 F	57.0	Nomegestrol acetate (1); endometriosis	15	Yes	Left	Yes; SOM Dx
Malaize et al., 2021 (29)	Retrospective cohort	1 F	49.0	Nomegestrol acetate and chlormadinone acetate (1); uterine fibroid	10	Yes	Right	Yes; SOM Dx
Weill et al., 2021 (30)	Prospective cohort	7 F	50.1^c^	Cyproterone acetate (7); varied	18	NS	NS	Yes; SOM Dx
Voormolen et al., 2021 (31)	Retrospective cohort	85 F	48.1^c^	Cyproterone acetate (85); varied	NS	Yes	NS	Yes; NS
Malueka et al., 2022 (32)	Retrospective cohort	57 F	47.9	NS (46); contraception	NS	NS	NS	NS
Florea et al., 2023 (33)	Retrospective cohort	15 F	49.5	Cyproterone acetate (5), cyproterone acetate plus estrogen (5), nomegestrol acetate (3), nomegestrol acetate to cyproterone acetate (1), nomegestrol acetate to chlormadinone acetate (1); varied	16.8	Yes	Unilateral (13), bilateral (2)	Yes; SOM Dx
Planty-Bonjour et al., 2024 (4)	Retrospective cohort	13 F	50.2	Nomegestrol acetate (8), cyproterone acetate (2), chlormadinone acetate (2), progestogenic therapies, and nafarelin (1); varied	13.3	Yes (5)	Bilateral	Yes; SOM Dx
Porto et al., 2025 (13)	Retrospective cohort	53	51.9^c^	NS (53); contraception	>10	NS	Unilateral (82), bilateral (7)	NS
Steinmetz et al., 2025 (12)	Case report	1 F	42.0	Nomegestrol acetate (1); dysmenorrhea and menorrhagia	8	Yes	Right	Yes; SOM Dx

Dx, diagnosis; F, female; M, male; NS, not specified or uncertain relationship to SOMs; postop, postoperatively; SOM, spheno-orbital meningioma.

a More patients are documented in the studies than those described in this table. Only patients with both a confirmed diagnosis of SOM and a known history of hormone therapy were included in the analysis. Mean patient ages and length of hormone therapy use were adjusted to reflect only those included in the analysis when possible.

b The mean age of patients in the study by Apra et al. was manually calculated to reflect that of the cohort.

c The mean ages depicted are not representative of patients with both a diagnosis of SOM and a history of hormone therapy, but rather of the whole cohort, because the information was not available. Therefore, the mean age at diagnosis in these instances includes patients with meningiomas in general, rather than those with the SOM subtype.

**Table 2:** Treatment methodologies, follow-up durations, and tumor behaviors in studies examining the association between spheno-orbital meningiomas and hormone therapy^a^.

Author	Treatment	Mean time to F/U, mo	Meningioma behavior (tissue vs bone)	Key finding
Apra et al., 2020 (22)	Surgery	NS	NS	Endogenous and exogenous progesterone have a role in SOM growth. SOMs in women had more PRs than those in men.
AbiJaoude et al., 2021 (28)	Conservative	12	Decrease in soft tissue; stability in intraosseous	Despite soft tissue behavior, the intraosseous component continues to grow after hormone therapy cessation.
Malaize et al., 2021 (29)	Surgery	36	Decrease in size (not specific to soft tissue or intraosseous)	Decrease in 30% of progestin-associated meningiomas after hormone therapy cessation. Not specific to SOM.
Weill et al., 2021 (30)	NS	30	Decrease in size (not specific to soft tissue or intraosseous)	Decrease (72% of cases) or stabilization (20% of cases) in meningioma volume after hormone therapy cessation. Not specific to SOM.
Voormolen et a.l, 2021 (31)	Surgery plus radiation therapy	NS	NS	SOMs appear to be specific to cyproterone acetate; risk of developing a meningioma while receiving cyproterone acetate decreased 73% after 1 y of hormone therapy cessation (a decline in aHR from 6.6 (95% CI, 4.0–11.1) to 1.8 (95% CI, 1.0–3.2). Not specific to SOM.
Malueka et al., 2022 (32)	Surgery	NS	NS	Patients with a history of hormone therapy had a higher proportion of SOMs than patients without hormone therapy (OR, 2.57, p=0.04).
Florea et al., 2023 (33)	Conservative	23	Decrease in soft tissue; increase (14) or decrease (1) in intraosseous	Decrease or stabilization of soft tissue after hormone therapy cessation; increased rate of intraosseous progression after hormone therapy cessation in patients with a history of cyproterone acetate plus estrogen (RR, 1.94, p=0.02).
Planty-Bonjour et al., 2024 (4)	Surgery (11), conservative (1), hybrid^b^ (1)	Varied	Decrease in soft tissue after conservative treatment	SOMs have a high hormonal dependence.
Porto et al., 2025 (13)	Surgery	78	NS	Postoperative soft tissue volume is associated with significantly higher odds of recurrence (aOR, 1.34, 95% CI, 1.13–1.59; p<0.001). A history of >10 y of hormone therapy also has higher odds of recurrence (not statistically significant; OR, 1.99, 95% CI, 0.69–5.79; p=0.21).
Steinmetz et al., 2025 (12)	Surgery	3	Decrease in soft tissue; increase in intraosseous	Rate of SOM proliferation decreases after hormone therapy cessation. Continued intraosseous growth unrelated to proliferation rate.

aOR, adjusted odds ratio; aHR, adjusted hazard ratio; F/U, follow-up; NS, not specified or uncertain relationship to SOMs; OR, odds ratio; PR, progesterone receptor; RR, relative risk; SOM, spheno-orbital meningioma.

a Mean time to follow-up was adjusted to reflect only those included in the analysis when possible. Conservative therapy was defined as hormone therapy cessation with radiologic follow-up to assess tumor volumetric changes. SOM component behavior was defined as the changes in tumor volume relative to the soft tissue and intraosseous components during follow-up after treatment.

b Hybrid therapy was used by Planty-Bonjour et al. for a patient with a bilateral SOM in whom conservative treatment was given for the right SOM and surgery was performed for the left.

**Figure 2:**
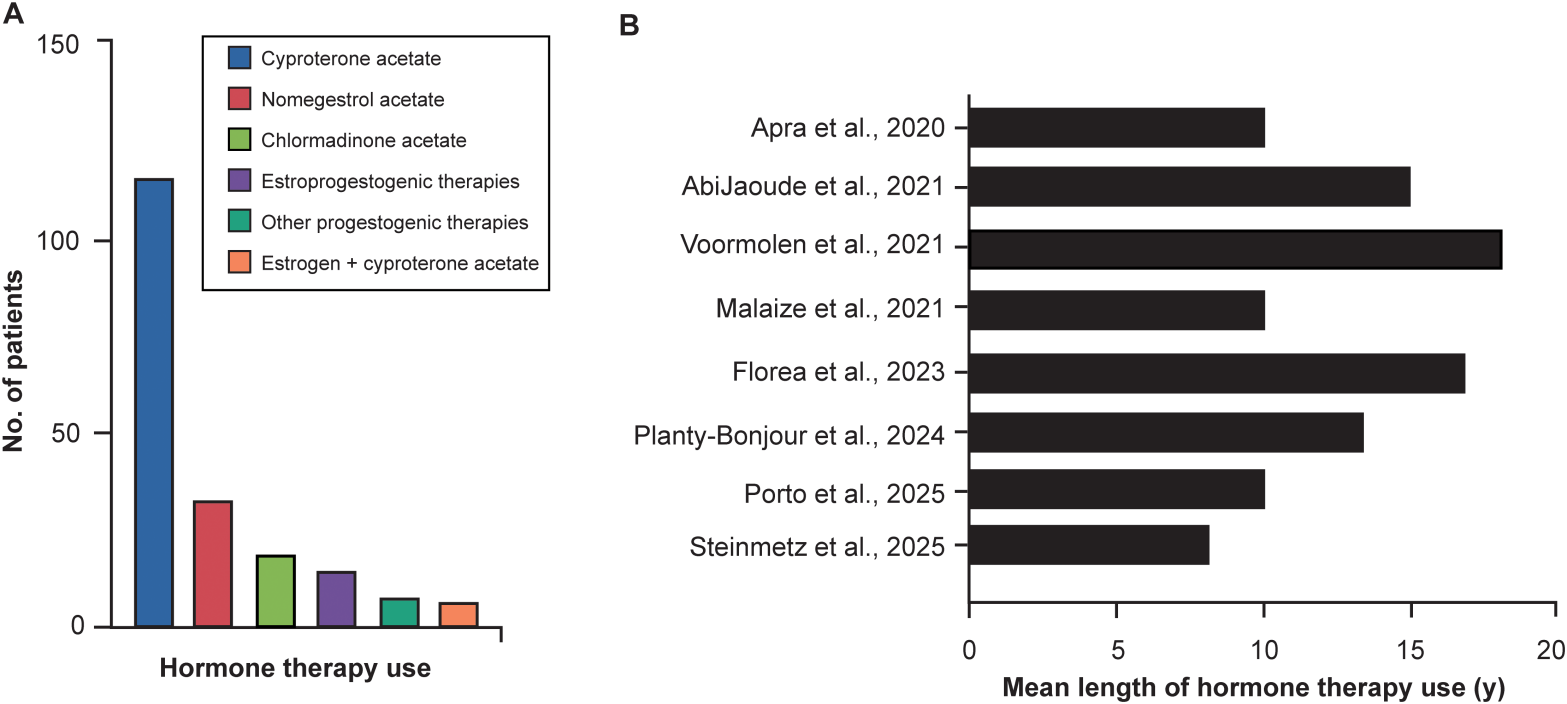
Trends in hormone therapy use and recent publications on its relation to spheno-orbital meningiomas (SOMs). Data were extrapolated from **Tables 1** and **2**. **(A)** Number of patients with a history of hormone therapy and the specific therapies they used. Each column includes patients with a history of more than 1 therapy and those who switched therapies over time. **(B)** Patients’ mean length of hormone therapy before SOM diagnosis for each study. When possible, the mean length of hormone therapy use listed in each study was adjusted to reflect only those patients included in our review (4, 12, 13, 22, 28, 29, 31, 33). Because Porto et al. (13) described only patients with a history of hormone therapy use for more than 10 years, 10 was used as the mean length of use for that study. Only studies with clear data on the mean length of hormone therapy among patients with SOMs were included. Used with permission from Barrow Neurological Institute, Phoenix, Arizona.

In the 6 studies with available data on tumor laterality, 98 patients had unilateral tumors and 22 had bilateral tumors. All studies reported that patients had a documented history of hormone therapy before the SOM diagnosis. The specific agents used in these studies are shown in **Figure 2A**. Cyproterone acetate was the most commonly used hormone therapy in the studies that reported specific medications (4, 12, 13, 22, 28–31, 33), with a total of 114 patients. An estrogen derivative in association with cyproterone acetate (33) was the least frequently reported, with only 5 recipients. The remaining therapies were as follows: nomegestrol acetate, 31 patients; chlormadinone acetate, 17; estroprogestogenic therapies, 13; and other progestogenic therapies, 6 (**Figure 2A**). When available, the duration of hormone therapy ranged from 8 to 18 years, with a mean (SD) of 12.6 (3.67) years (**Figure 2B**) (4, 22, 28, 29, 31, 33).

Information on the cessation of hormone therapy was not specified in all studies; however, of the 7 studies with available data, hormone therapy was ceased after either diagnosis or surgical resection (4, 12, 22, 28, 29, 31, 33). The PR status of the SOM was reported in only 3 studies, with positive expression observed in all patients tested (4, 12, 22).

SOMs frequently showed both soft tissue and osseous components. Six of the 10 articles reported a decrease in the SOM volume after hormone therapy cessation (4, 12, 28, 29, 31, 33). Two of these 6 studies (29, 31) evaluated overall tumor size without specifying involvement of specific components, whereas another study (4) focused on the soft tissue portion of the tumor. In the remaining 3 of these 6 studies, volume changes in the soft tissue and intraosseous components were measured separately (12, 28, 33). All patients in these 3 studies had decreased soft tissue volume, whereas the intraosseous component increased in all but 1 patient, who experienced a reduction in both components.

Surgical treatment was reported in 7 of the 10 studies (4, 12, 13, 22, 29, 30, 32). Gross total resection was achieved in select cases; however, subtotal resection was more common because of the tumor’s location and involvement of critical structures. Recurrence was reported in subtotal resection cases, with follow-up durations ranging from 3 months to more than 6 years. Conservative management, defined as hormone therapy cessation with radiologic surveillance, was reported in 3 studies (4, 28, 33). In all 3 of the patients, the soft tissue component of the tumor decreased in volume on radiologic images.

Across all studies that reported tumor classification, the meningiomas were predominantly classified as World Health Organization (WHO) grade 1 or 2 (4, 12, 13, 28, 29, 31, 32). One study reported a small subset of 5 tumors classified as ‘other,’ which were not further specified and may represent WHO grade 2 or 3 (32). Proptosis, headaches, and visual symptoms were the most frequently reported symptoms. Details about radiation therapy were limited. Five articles mentioned the use of adjuvant radiation therapy or radiosurgery (4, 13, 29–31); however, none reported patient data specific to SOMs.

## 4 Discussion

**Figure 3:**
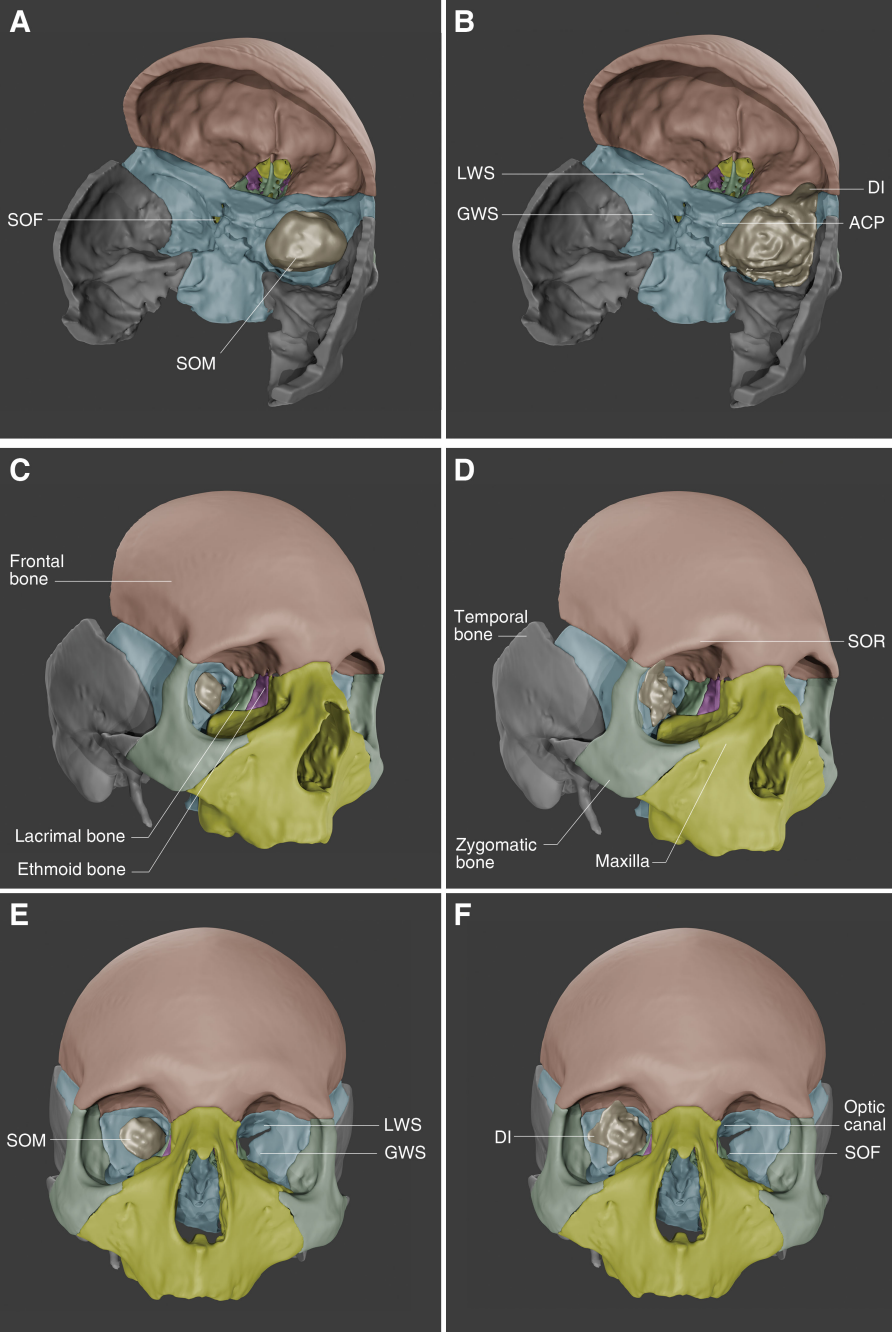
Three-dimensional (3D) reconstructions of spheno-orbital meningiomas (SOMs) with and without osseous invasion: **(A, B)** Superolateral oblique view showing the middle cranial fossa with the SOM, demonstrating its bulk in relation to the skull base **(A)** without apparent extension into adjacent bone and **(B)** with dural and bony extensions infiltrating the middle cranial fossa and adjacent spheno-orbital bones. **(C, D)** Anterolateral view depicting the intraorbital component of the tumor near the periorbital structures **(C)** without visible bony involvement and **(D)** with direct involvement of the orbital wall structures. **(E, F)** Anterior view highlighting **(E)** the spatial relationship between the tumor bulk, orbit, and surrounding craniofacial bones and **(F)** the invasive spread of the tumor into craniofacial bones, altering normal bony architecture. Bone and tumor segmentations were performed in 3D Slicer (https://www.slicer.org/) by tracing key cranial sutures for anatomical accuracy. Final 3D rendering and visualizations were completed in Blender (https://www.blender.org/). ACP, anterior clinoid process; DI, dural involvement (of a SOM); GWS, greater wing of the sphenoid bone; LWS, lesser wing of the sphenoid bone; SOF, superior orbital fissure; SOR, supraorbital rim. Used with permission from Barrow Neurological Institute, Phoenix, Arizona.

Meningiomas can be challenging to manage when located at the skull base, and SOMs, recognized as a distinct skull-base subtype, demonstrate complex pathophysiology and demanding operative management (13, 35). Given their intimate involvement with the orbital apex, optic canal, and superior orbital fissure, SOMs can create substantial complications that affect visual acuity (**Figure 3**). Patients commonly experience a combination of proptosis, visual impairment, and ocular paresis (6). These clinical and anatomical complexities have primarily driven a surgical focus in managing and treating SOMs. However, recent literature suggests an association between hormone therapy and the growth of SOMs, which could have major implications for both tumor development and treatment strategies.

### 4.1 Hormone therapy as a risk factor for SOMs: evidence and controversies

**Figure 4:**
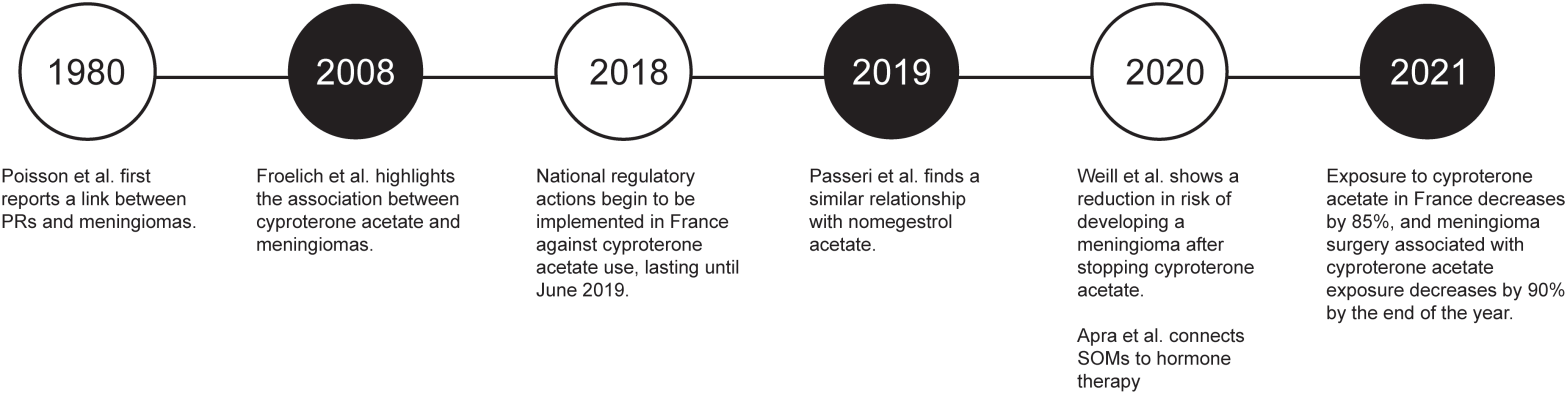
Timeline of key events in understanding the role of hormone therapy in meningiomas (22, 30, 37, 38, 40–42). The timeline was created in BioRender (https://www.biorender.com). PR, progesterone receptor; SOM, spheno-orbital meningioma. Used with permission from Barrow Neurological Institute, Phoenix, Arizona.

Within the past 5 years, interest has grown in the relationship between hormone therapy (particularly progestins) and SOMs. Although the cause of this growing interest in such a rare tumor remains uncertain, a possible reason may be the earlier proliferation of literature that emerged from France. The first reported link between PRs and meningiomas was a 1980 French study, which found 100% PR positivity in the cases studied (36, 37). In 2008, Froelich et al. (38) reported that hormone therapy, specifically cyproterone acetate, promotes growth in meningiomas and that hormone therapy cessation can cause tumor regression. This report led to further exploration of cyproterone acetate in 2015 (39), a similar report on nomegestrol acetate in 2019 (40), and an extensive study of more than 250,000 women receiving cyproterone acetate from 2007 to 2015 (30, 41). All the results showed either tumor growth (39, 40) or a significantly increased risk (30, 41) of developing meningiomas, with a notable decrease in these metrics after hormone therapy cessation. In 2020, Apra et al. (22) made the first connection between hormone therapy and SOMs in particular. Finally, after national regulatory actions were implemented in August 2018 and June 2019, exposure to cyproterone acetate had decreased by 85% at the end of 2021, and the number of meningioma operations required because of associations with cyproterone acetate had decreased by 90% (93% for women and 86% for men) (**Figure 4**) (22, 30, 37, 38, 40–42).

In the United States, hormone therapy use among postmenopausal women also decreased, from 26.9% in 1999 to 4.7% in 2020, with progesterone-only formulations accounting for just 10.5% of the hormone therapies used by the end of 2020 (43). Furthermore, the US Preventive Services Task Force in 2022 recommended against hormone therapy with combined estrogen and progestin for the primary prevention of chronic conditions in postmenopausal women (44). These changes may reduce the incidence of SOM in the future.

Although only 1 prospective study was found for this review, associations are being observed retrospectively regarding hormone therapy as both a risk factor and a modulatory factor for SOMs (4, 12, 13, 22, 28, 29, 31–33). Malueka et al. (32) found that SOM was significantly more likely to develop in patients with a history of hormonal contraceptive use (odds ratio, 2.57; 95% CI, 1.04–6.37; p=0.04). Moreover, the duration of hormone therapy use also appears to influence the risk of developing SOMs (16). In our review, the mean length of hormone therapy use at diagnosis was 12.6 (3.6) years, which aligns with the findings of Andersen et al., who reported an odds ratio of 1.7 (95% CI, 1.2–2.3) for developing a meningioma after 10 years of hormone therapy use (16). Furthermore, Weill et al. claimed that the risk of meningiomas during hormone therapy was dose dependent (30, 41). Their study showed that patients with a cumulative exposure of at least 3 g of cyproterone acetate had a 6.6-fold higher hazard ratio (95% CI, 4.0–11.1) of a meningioma developing compared with patients with less cyproterone acetate exposure.

Although the biological mechanism underlying the association between hormone therapy and meningiomas is not yet fully understood, studies have shown that meningiomas of the spheno-orbital region have a high PR expression (45), leading to increased proliferation and tumor growth (4, 22). Planty-Bonjour et al. (4) found that hormone therapy was not statistically significantly associated with the clinical manifestations of SOMs, such as visual impairment or proptosis, but 91% of SOMs had a high PR expression, between 70% and 100% positivity.

Recent literature suggests that this progesterone-driven proliferation may be influenced by steroid receptor coactivators: steroid receptor coactivator-1 (SRC-1), transcriptional intermediary factor 2 (TIF2/SRC-2), and amplified in breast cancer 1 (AIB1/SRC-3) (19, 20). In meningiomas, a high expression of these coactivators has been reported, with SRC-1 present in 81% of cases and both SRC-2 and SRC-3 found in 76%. These proteins do not bind DNA directly but interact with nuclear hormone receptors such as PR to increase the transcription of growth-related genes. SRC-3 has been linked to both the development and progression of meningiomas, further supporting its potential role in modulating tumor behavior in hormonally sensitive subtypes such as SOMs. Interestingly, SRC-1 and SRC-2 appear to have a strong connection with the expression of PR but not with the expression of estrogen receptors, which suggests these steroid receptors coactivators may play a more specific role in progesterone signaling. This possibility could help explain why progestins, rather than estrogens, seemed to have a bigger effect on SOM growth in the patients in our review, in which most patients reporting SOMs and a history of hormone therapy were receiving some form of progesterone analog (19, 20).

Notably, not all study findings agree regarding the association of the level of PR expression and its overall effect. Maiuri et al. found that lateral skull base tumors, including SOMs, have less PR positivity than medial skull base tumors (18). Furthermore, Kuroi et al. suggested that although skull base meningiomas have higher PR expression than non–skull base meningiomas, this elevated expression is associated with a better prognosis, which contradicts previous studies mentioned (45). These inconsistencies underscore the need for further research to clarify the role of PR expression and better define the mechanisms by which hormone therapy influences tumor development and growth in this anatomical region.

### 4.2 Tumor response after hormone therapy cessation

In our review, 6 articles reported a regression in tumor volume after cessation of hormone therapy (4, 12, 28, 29, 31, 33). In a novel prospective study on the effects of cyproterone acetate cessation in meningiomas, including 7 cases of SOMs, Voormolen et al. (31) found a volume decrease in 72% of the meningiomas tested, with another 20% having stabilized. This decrease in volume, however, does not appear to be uniform across the anatomy of SOMs. Three of the 6 studies yielded opposing results regarding the dual nature of SOMs. In a case report, a 57-year-old woman with a 15-year history of receiving nomegestrol acetate had a decrease in the soft tissue component of her SOM with stability in the osseous component 1 year after hormone therapy cessation (28). Another study, which described a 42-year-old woman with an 8-year history of nomegestrol acetate therapy, reported soft tissue regression with intraosseous growth after hormone therapy cessation (12). The results of the remaining study continued with this trend in its cohort, showing a decrease in the soft tissue portion of all their SOM cases, with continued intraosseous growth in 93% (33). These discovered outcomes highlight a critical question in the treatment of these tumors: can SOMs be managed, at least in part, nonoperatively with serial clinical and radiographic surveillance after cessation of hormone therapy?

### 4.3 Balancing conservative and surgical strategies in SOM treatment

**Figure 5:**
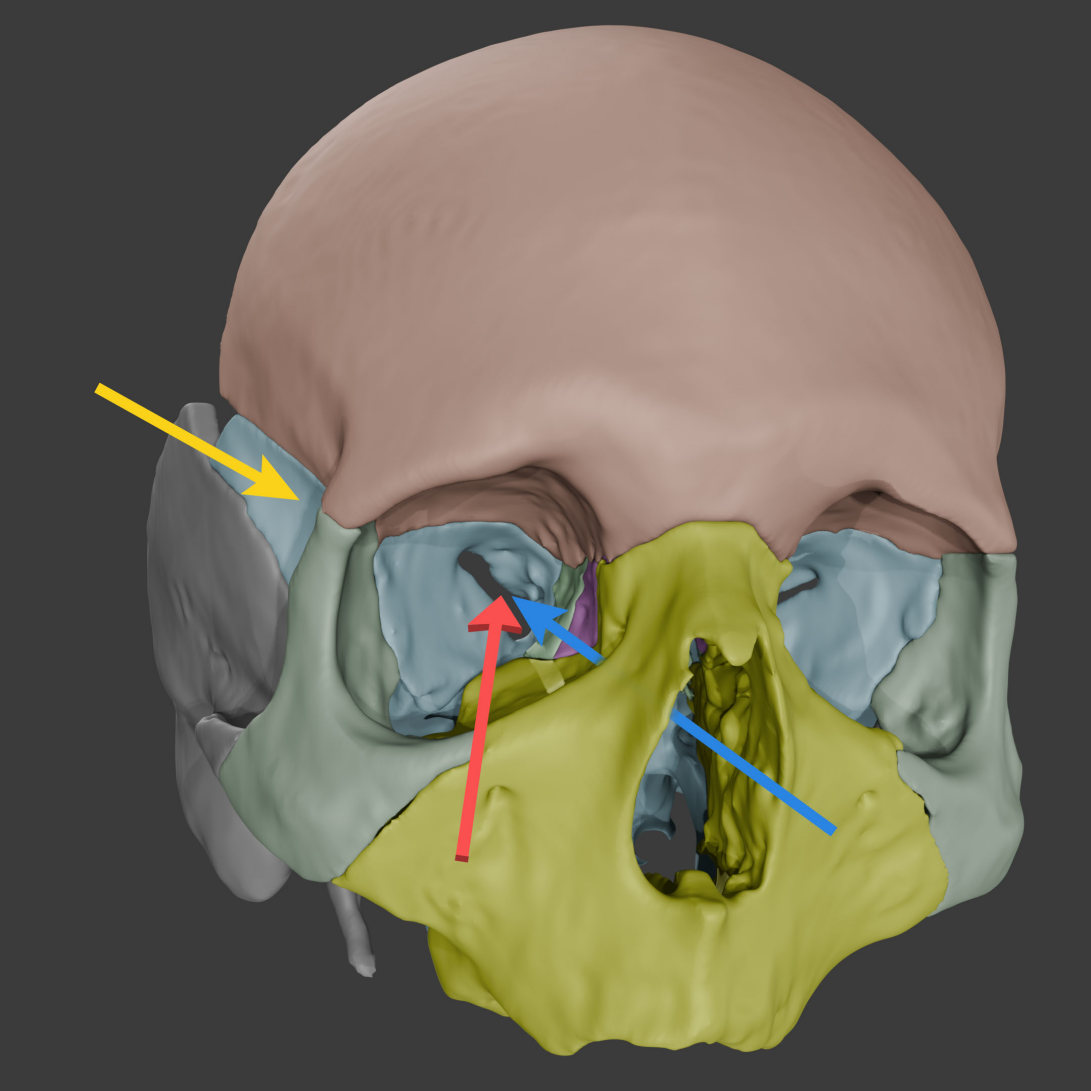
Three-dimensional (3D) reconstructions of different surgical approaches for spheno-orbital meningiomas. Access routes are indicated for the pterional transcranial (*yellow arrow*), endoscopic transorbital (*red arrow*), and endoscopic endonasal (*blue arrow*) surgical techniques. Bone segmentations were performed in 3D Slicer (https://www.slicer.org/) by tracing key cranial sutures for anatomical accuracy. Final 3D rendering and visualizations were completed in Blender (https://www.blender.org/). Used with permission from Barrow Neurological Institute, Phoenix, Arizona.

**Figure 6:**
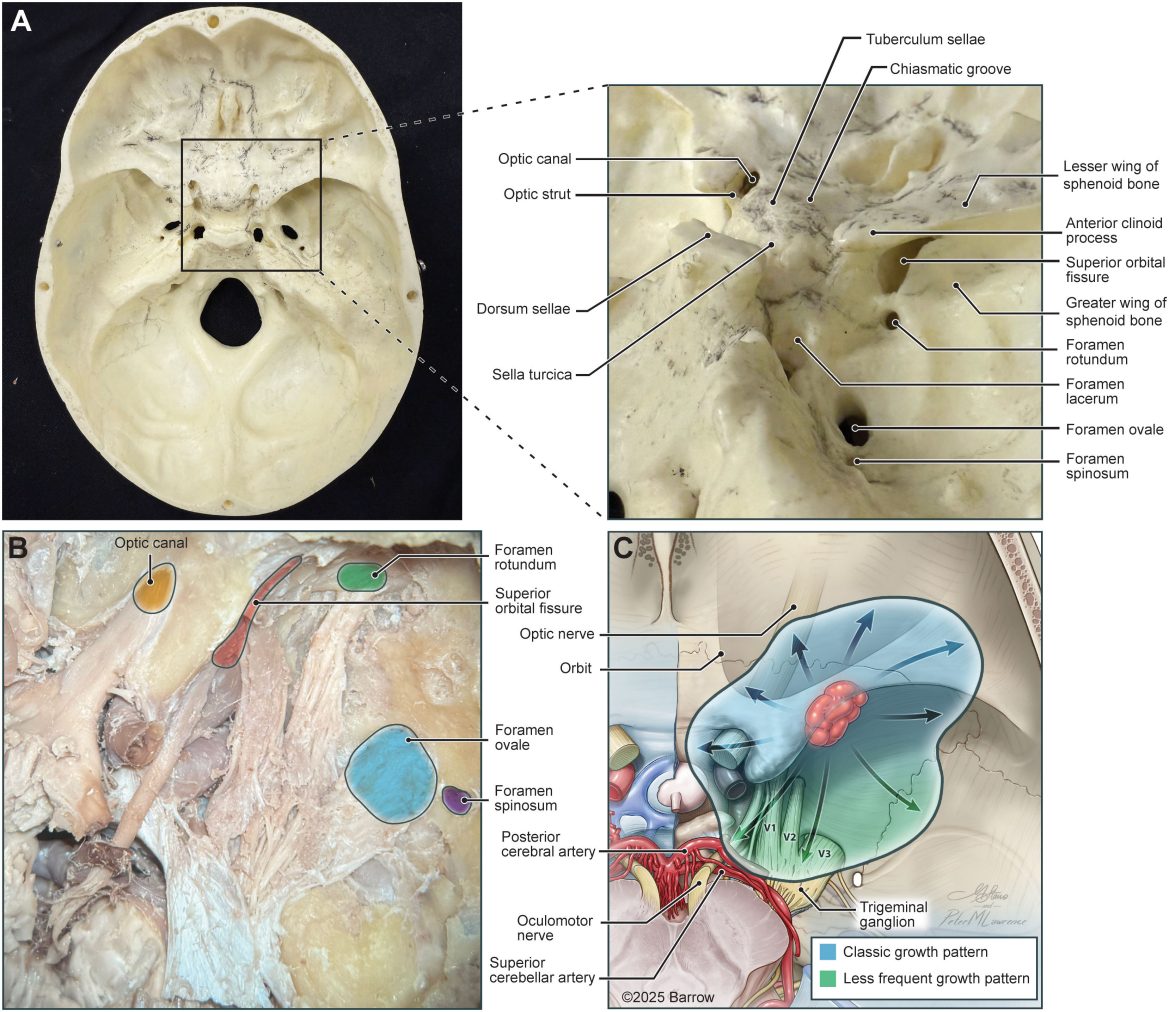
Anatomical correlates in the growth and development of spheno-orbital meningiomas (SOMs) depicted with models, illustrations, and cadaveric dissections: **(A)** Axial view of a skull base model emphasizing an oblique view of the middle cranial fossa and included foramina (*inset*). **(B)** Oblique view of a cadaveric dissection after dural removal, highlighting the optic canal (*orange*), superior orbital fissure (*red*), foramen rotundum (*green*), foramen ovale (*blue*), and foramen spinosum (*purple*). **(C)** Illustration of a superior view of the middle cranial fossa highlighting a common point of origin of SOMs (*red lesion*) as well as potential areas of soft tissue and intraosseous growth (*blue and green shading*). Used with permission from Barrow Neurological Institute, Phoenix, Arizona.

The standard treatment of SOMs has been surgical resection, with an emphasis on symptom relief rather than a cure (8). Among the various options available to surgeons, the most common approach appears to be a microsurgical transcranial approach, likely involving a pterional craniotomy with extradural drilling of the hyperostotic sphenoid bone and associated orbital wall (**Figure 5**) (2, 9–12). Intradural tumor resection and optic nerve decompression follow this approach when warranted. The approach provides various outlets to different portions of SOMs, including the cavernous sinus, superior orbital fissure, and orbital apex (**Figure 6**) (9).

**Figure 7:**
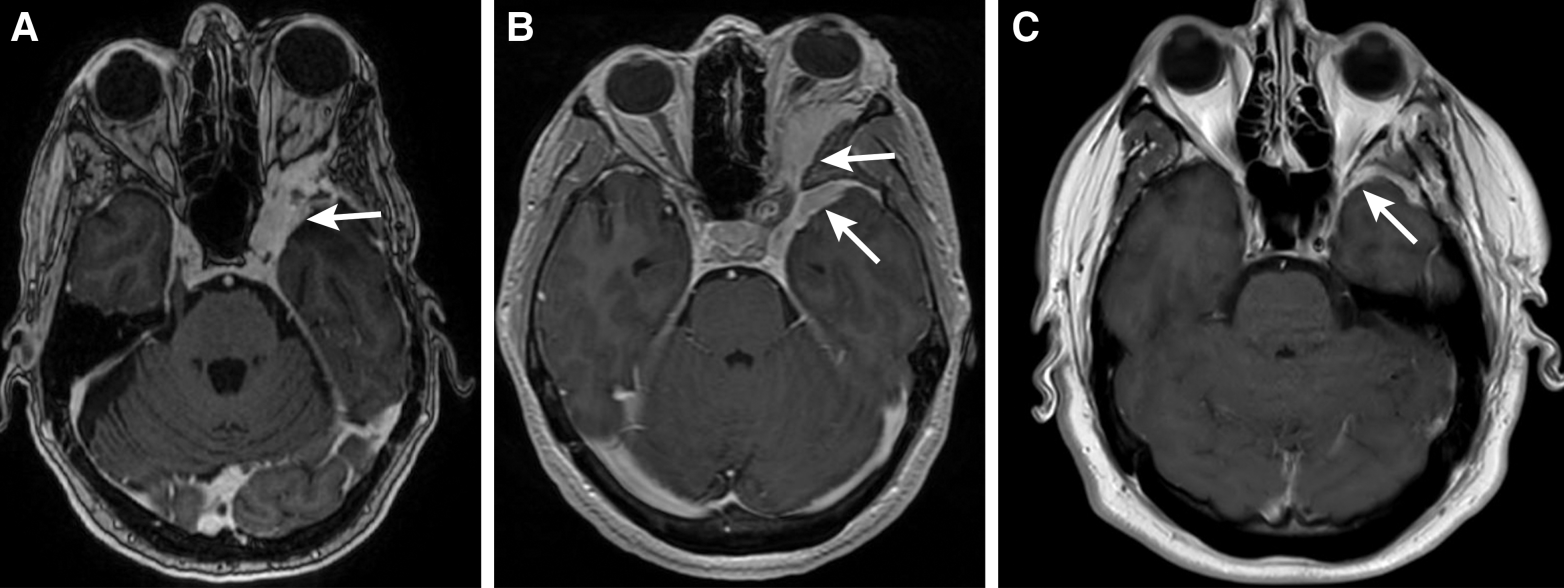
Representative axial T1-weighted contrast-enhanced magnetic resonance images of the brain from 3 patients with a spheno-orbital meningioma (SOM): **(A)** SOM invading the left orbital apex (*arrow*), with an extension into the orbit causing proptosis. **(B)** A left-sided SOM showing dural thickening and signal enhancement, with progressive growth toward the orbit (*arrows*). **(C)** A small left-sided SOM showing dural thickening and increased signal intensity, without evidence of intraorbital extension (*arrow*). Used with permission from Barrow Neurological Institute, Phoenix, Arizona.

Unfortunately, the success rate of attaining gross total resection largely depends on the extent of soft tissue invasion of the cavernous sinus or superior orbital fissure because attempted resection could increase the chances of morbidity and cranial nerve deficits (**Figure 7**) (9). These tumors have a higher recurrence rate than other meningiomas, primarily when only subtotal resection is achieved (13, 46). When a portion of the tumor must be spared for the patient’s safety, recurrence is more likely if the residual component is soft tissue rather than bone, thus raising concerns about the long-term efficacy and clinical justification of subtotal resection in such scenarios (13). In particular, perioptic SOMs pose a unique surgical challenge because aggressive decompression or manipulation of the optic canal and orbital apex carry a risk of iatrogenic visual decline (47). This risk underscores the importance of careful patient selection and individualized surgical planning for treatment of lesions abutting the optic apparatus. Comprehensive preoperative and postoperative ophthalmologic evaluation should therefore be an integral component of management, guiding decision-making and outcome assessment.

Our review highlights recent findings that could help shape a new approach to treating SOMs in this selected patient group. Despite known regression, a purely conservative approach in treating SOMs via hormone therapy cessation and monitoring might not be optimal when osseous involvement has occurred. The literature does not indicate any significant reduction in intraosseous tumor volume after hormone therapy cessation alone (4, 12, 28, 33). Although soft tissue regression would alleviate some symptoms, the osseous component still can pose a threat to adjacent structures, such as the optic nerve. More likely, hormone therapy cessation should be part of a hybrid approach to treating SOMs in conjunction with surgical resection. Delaying surgery with the initial, conservative therapy of hormone therapy cessation can allow time for the soft tissue aspect to regress.

With a smaller, more defined osseous remnant of the tumor, surgical intervention can be performed with a lower risk of injuring the cavernous sinus or the superior orbital fissure. In this context, PR antagonists could be considered part of this hybrid approach in patients not actively receiving exogenous hormone therapy. One case series involving intracranial meningiomas reported a case of SOM with both clinical improvement and a 22% reduction in tumor volume after treatment with mifepristone, a PR antagonist (48). However, the patient described was postmenopausal and had recently discontinued hormone replacement therapy. This raises the possibility that the tumor regression was influenced by the cessation of an exogenous hormone therapy and the natural decline in endogenous hormone levels during menopause rather than by PR antagonism alone. Consistent with this limitation, *in vivo* studies evaluating PR antagonists in meningioma treatment have reported mixed results, underscoring the need for further investigation before their role in SOM management can be clearly defined (34).

Additionally, minimally invasive approaches, such as the endoscopic transorbital approach, can be considered in patients with a smaller intracranial extension after hormone therapy cessation (**Figure 5**). Although its effectiveness in resecting the soft tissue component remains limited and controversial, the endoscopic transorbital approach has shown utility in SOMs with predominantly osseous involvement (9, 49–52).

However, it is worth noting that intraosseous growth after hormone therapy cessation can be self-limited and does not necessarily result in visual impairment, thus negating the need for any immediate surgical intervention (53). This pattern of self-limiting bony progression may be interpreted as a form of reactive hyperostosis, a phenomenon described in other inflammatory and tumoral conditions, representing a stimulatory response rather than direct neoplastic invasion (54, 55). However, this theory has been questioned within the context of meningiomas (56, 57). Historically, concern for intraosseous growth and the risk of visual compromise has led to surgical intervention in patients with SOMs. Nevertheless, emerging evidence indicates that, in many such cases, visual deterioration does not occur, and associated manifestations, such as proptosis, may also remain stable or regress over time (33, 53). Notably, Florea et al. reported that, despite ongoing osseous progression in select patients, almost none experienced visual decline, supporting a more conservative and individualized management strategy (33).

Ultimately, the involvement of PRs and their association with hormone therapy in SOMs presents an opportunity to investigate more conservative treatment options for these patients. Additional studies should be conducted to determine the efficacy of these approaches in managing SOMs.

### 4.4 Future directions

With the evidence from our review supporting the involvement of hormone therapy exposure in the prognosis of SOMs, a well-documented history of hormone therapy exposure should be routinely integrated into patient evaluation and treatment planning. Although no SOM-specific management guidelines exist, recommendations addressing meningiomas in the context of sex hormone exposure are likely applicable to this subgroup and provide a general clinical framework. However, these guidelines do not specifically account for the distinct anatomical involvement, hyperostotic behavior, and surgical complexity that are characteristic of SOMs.

As a result, several clinically relevant questions are largely unanswered and understudied in patients with SOMs, including when to cease hormone therapy, how long to observe a patient after hormone therapy cessation before resection, when conservative management is appropriate, and in which patient populations conservative management should be used. Prospective studies would yield beneficial data on the prognosis of SOMs after hormone therapy cessation, highlighting the extent of regression, soft tissue and intraosseous differences, and clinical correlates. Furthermore, there is an opportunity to evaluate the outcomes and rationale for combining hormone therapy cessation with surgical resection as a hybrid management approach for patients with SOMs. Other potential studies could include the use of antiprogestin therapy in selected patients.

In recent years, the growing integration of artificial intelligence and deep learning algorithms into neurosurgery has opened new avenues for personalized care; similarly, the development of patient-specific predictive models for hormone therapy, already explored in other cancers such as prostate cancer, may hold promising potential for advancing individualized management strategies in SOMs (58–61).

### 4.5 Limitations

Because of the heterogeneity of the available literature in our review, performing a quantitative meta-analysis was not feasible. However, as the body of literature grows, future meta-analyses may become possible to assess the association between hormone therapy and SOMs definitively. The included studies also had limitations. All but 1 of these studies were retrospective, which may have introduced potential selection bias into their conclusions. Seven of our studies introduced surgery as a confounding variable, making it difficult to separate the success of hormone therapy cessation from that of resection (4, 12, 13, 22, 29, 30, 32).

Furthermore, although every study in our review included data on SOMs, the larger focus of 5 included studies was on the broader class of meningiomas rather than SOMs (28–31, 33). Because of this situation, not all studies emphasized the distinct regression habits of the soft tissue and osseous tumor portions associated with SOMs. In the studies that highlighted volumetric changes in the soft tissue and osseous components, regression was defined differently, and no standardized threshold or timeframe was indicated.

## 5 Conclusions

This systematic literature review highlights the emerging association between hormone therapy, particularly progestins, and the risk and progression of SOMs. Although existing studies support hormone therapy as a risk factor and suggest that cessation can lead to regression of the soft tissue component, evidence remains limited and inconsistent, particularly concerning intraosseous involvement. The variability in outcomes underscores the complexity of SOM pathophysiology and the need for nuanced treatment approaches.

In the context of progestin-induced meningiomas, an initial conservative approach of watchful waiting after hormone therapy cessation is increasingly recommended, particularly in patients with preserved visual function. Similarly, in selected SOM patients without exophthalmos, visual field deficits, or visual acuity loss, a conservative approach can be considered. Furthermore, the role of adjunctive strategies such as PR antagonist therapy remains investigational, with existing evidence confounded by hormone withdrawal and perimenopausal hormonal changes.

Currently, no standardized guidelines exist for the management of SOMs in the context of hormone therapy, presenting an opportunity for novel studies and a deeper analysis of this association and its implications in clinical management. Further prospective studies are necessary to determine the optimal timing, indications, and efficacy of hormone therapy cessation and hybrid approaches that combine medical and surgical therapies. Establishing standardized metrics for regression and recurrence will also be critical in guiding future clinical decision-making.

## Data Availability

The original contributions presented in the study are included in the article/**Supplementary Material**. Further inquiries can be directed to the corresponding author.
