# Supplementary material for: Association of hormone therapy with spheno-orbital meningiomas: bridging evidence and unknowns
Source: Front Oncol. 2026 Mar 16;16:1764350. doi: 10.3389/fonc.2026.1764350 (PMC13033496; doi:10.3389/fonc.2026.1764350)
Supplement: Supplementary file 1 [file Table1.docx]

**Supplemental Table 1.** JBI risk of bias quality assessment^a^

| **Study** | **Q1** | **Q2** | **Q3** | **Q4** | **Q5** | **Q6** | **Q7** | **Q8** | **Q9** | **Q10** | **Q11** | **Yes Rate (%)** | **Risk** |
| --- | --- | --- | --- | --- | --- | --- | --- | --- | --- | --- | --- | --- | --- |
| Apra et al, 2020 (21) | U | Y | N | Y | N | N | Y | Y | NA | NA | Y | 56% | Mod |
| AbiJaoude et al, 2021 (27) | Y | Y | Y | Y | Y | Y | Y | Y | – | – | – | 100% | Low |
| Malaize et al, 2021 (28) | NA | NA | Y | U | N | N | Y | Y | Y | NA | Y | 63% | Mod |
| Weill et al, 2021 (29) | Y | Y | Y | Y | Y | Y | Y | Y | Y | Y | Y | 100% | Low |
| Voormolen et al, 2021 (30) | NA | NA | Y | U | U | N | Y | Y | N | N | Y | 44% | High |
| Malueka et al, 2022 (31) | Y | Y | N | Y | Y | N | Y | U | NA | NA | Y | 67% | Mod |
| Florea et al, 2023 (32) | Y | NA | Y | Y | N | NA | Y | Y | Y | U | Y | 78% | Low |
| Planty-Bonjour et al, 2024 (4) | Y | Y | Y | Y | N | N | Y | Y | Y | N | Y | 73% | Low |
| Porto et al, 2025 (13) | Y | Y | Y | Y | Y | Y | Y | Y | U | U | Y | 82% | Low |
| Steinmetz et al, 2025 (12) | Y | Y | Y | Y | Y | Y | Y | Y | – | – | – | 100% | Low |

Abbreviations: JBI, Joanna Briggs Institute; Mod, moderate; N, no; NA, not applicable; Y, yes.

^a^ Q1 through Q11 refer to questions 1 through a possible 11 from the respective JBI Critical Appraisal Checklist, which applies to either case reports or cohorts, depending on the specific study. The risk of bias was rated as low if 70% or more of the checklist items were met, moderate if 50% to 69% were met, and high if fewer than 50% were met.
